# Evaluation of nitrate reductase assay in 7H11 agar for diagnosis of multi-drug-resistant tuberculosis in eastern Nepal

**DOI:** 10.1186/s41182-018-0109-6

**Published:** 2018-07-25

**Authors:** Dristi Halwai, Rajendra Gurung, Nimesh Poudyal, Dharanidhar Baral, Shyamal Kumar Bhattacharya

**Affiliations:** 1 Department of Microbiology, B.P. Koirala Institute of Health Sciences, Dharan, Nepal; 2 Department of Community Medicine & School of Public Health, B.P. Koirala Institute Health Sciences, Dharan, Nepal

**Keywords:** Isoniazid, Nitrate reductase assay, Rifampicin, Tuberculosis, 7H11 agar

## Abstract

**Background:**

Emergence of multi-drug-resistant tuberculosis is a serious challenge for successful global tuberculosis control. Early diagnosis of drug-resistant tuberculosis by direct nitrate reductase assay (NRA) aids in appropriate treatment and reduction in disease transmission, particularly in countries with high tuberculosis burden. The aim of this study was to evaluate the performance of NRA for direct detection of resistance to rifampicin and isoniazid in *Mycobacterium tuberculosis* in laboratories with limited resources.

**Methods:**

Fifty-eight new smear-positive sputum samples were processed as per the guidelines of revised national tuberculosis control program, India. The performance of NRA on middlebrook 7H11 agar was evaluated for detection of rifampicin and isoniazid resistance directly on smear-positive sputum specimens, and the results were compared with conventional proportion method. Sensitivity and specificity of the test were compared with the gold standard proportion method. Mc Nemar chi-square test was used to find out the significant difference between two methods.

**Results:**

Direct NRA for detection of rifampicin resistance was 85.7% sensitive and 100% specific, whereas sensitivity and specificity of isoniazid resistance were 87.5% and 100%, respectively. Agreement between NRA and proportion method was 98% for both the drugs. The mean days of drug susceptibility testing results were 19.3 days for NRA and 72 days for conventional proportion method. The results of NRA were available in 21 days for 83% of the samples.

**Conclusions:**

Direct NRA on middlebrook 7H11 medium is a highly sensitive, reliable, and significantly faster method to perform drug susceptibility testing. It has the potential to be implemented for rapid detection of multi-drug-resistant tuberculosis against insufficient resources.

## Background

Tuberculosis (TB) is one of the leading causes of death and disability in low- and middle-income countries despite being curable [1]. The emergence of drug-resistant tuberculosis has further worsened the impact of this disease [2]. As per the results of a drug resistance survey conducted in Nepal, around 2.2% of new cases and 15.4% of retreatment cases had multi-drug-resistant TB (MDR-TB) among which 8% of the cases were found to be extensive drug-resistant tuberculosis (XDR-TB) [3].

In countries like Nepal, TB is still being diagnosed and treated on the basis of microscopy which has a low sensitivity of 50–60% [4]. Although conventional culture method is the gold standard method for diagnosis and drug susceptibility testing (DST), it requires 6–8 weeks to yield results [5]. It has been estimated that up to 96% of MDR-TB cases are not being diagnosed promptly and treated effectively [6].

For global control of drug-resistant TB, laboratories must strengthen the capacity to perform DST of first and second line drugs using rapid methods [7]. Microscopic observation of drug susceptibility (MODS) and nitrate reductase assay (NRA) have been recommended by the World Health Organization (WHO) as rapid and inexpensive methods for DST. MODS rely on microscopic observation of cord formation in microtitre plate containing liquid culture medium through an inverted microscope. It requires additional equipment, consumables, and a skilled laboratory personnel which is difficult to obtain in resource-limited settings [8].

NRA is based on the ability of *Mycobacterium tuberculosis* (MTB) to reduce nitrate incorporated in the medium to nitrite, which can be detected by the change in color by adding Griess reagent [2]. Direct NRA using middlebrook 7H11 agar is faster and reliable method to detect drug resistance in tuberculosis. It has shown convincing results with high sensitivity and specificity in a short turnaround time (TAT) [9].

The purpose of this study was to evaluate the performance of NRA as a rapid diagnostic technique for direct DST of *Mycobacterium tuberculosis* in a country with high TB burden and laboratories with limited resources.

## Methods

### Study design and setting

This laboratory-based comparative cross-sectional study was conducted in the Department of Microbiology, BPKIHS, Nepal. A total of 58 new smear-positive sputum samples received in the laboratory were included. Sample size calculation was done using N Master 2.0 version with power, 80%; confidence interval, 95%; sensitivity of reference test, 99%; sensitivity of new test, 87.5% [2]; and estimated sample size (*n*), 58.

### Culture and drug susceptibility testing on LJ medium

Sputum samples with the bacillary load of 1+ (1–9 bacilli/100 fields) and more were included in our study. Sputum samples were decontaminated by using modified Petroff’s method [10]. The sediments were retained for culture in middlebrook 7H11 media for NRA and Lowenstein Jensen (LJ) medium.

The sediments were inoculated into LJ medium and *para* nitro benzoic acid containing LJ medium (PNBLJ). The isolates were identified as MTB on the basis of slow growth rate, acid fast staining, absence of pigmentation, negative heat labile catalase test (68 °C), and no growth on PNBLJ [10].

All the procedures for DST were performed following standard guidelines issued by Ministry of Health and Family Welfare, New Delhi, India. Proportion method (PM) on LJ medium was used as the gold standard method for DST of tuberculosis [11].

*Mycobacterium tuberculosis* H37Rv reference strain was used as a quality control strain for both culture and DST.

### Nitrate reductase assay

#### Preparation and processing in NRA

NRA was performed on middlebrook 7H11 agar medium incorporated with potassium nitrate: 1000 μg/ml KNO_3_ was added in the medium. The media was then divided into three parts; one part containing 0.2 μg/ml of isoniazid (INH), one part containing 1.0 μg/ml of rifampicin (RIF), and one part without antibiotics used as the growth control. All three types of NRA bottles were labeled with a code. The drugs free and drugs containing NRA bottles were inoculated according to the standard procedure manual [9, 12, 13].

#### Interpretation of results-

0.5 ml of the Griess reagent was added to one drug-free NRA bottle on day 10. If there were any changes in color (light pink), then the corresponding antibiotic-containing bottles were tested for color development. If no color change was observed, then the procedure was repeated at day 14, 21, and finally at day 28. An isolate was considered resistant to a drug if the color in the drug containing bottle was greater than that in the drug-free medium.

*Mycobacterium intracellulare* strain was used as a negative control for NRA [14].

### Data analysis

The collected data was entered in Microsoft Excel-2010 and converted into SPSS version 11 for statistical analysis. For inferential statistics, sensitivity and specificity of the test with respect to the gold standard method were calculated. Mc Nemar chi-square test was used to find the significant difference between two methods.

## Results

Among the 58 samples processed, NRA detected growth in 53 samples and LJ medium detected growth in 52 samples. Sensitivity of growth detection by NRA and conventional method was 91.4 and 89.6% respectively with mean time of 19.2 days for NRA and 32.7 days for the conventional method. There was no significant difference in the sensitivity for growth detection by both the methods (*p* > 0.05).

Six sputum samples were excluded from our study as there were contamination and no growth on both the media. Fifty-two identical samples were included for the comparison of DST and TAT. The final susceptibility testing results for RIF and INH are depicted in Table 1.

**Table 1:** Comparison of nitrate reductase assay results with conventional proportion method

Drugs	Conventional PM	Nitrate reductase assay							
		Resistant	Sensitive	Sensitivity (95% CI)	Specificity (95% CI)	PPV (%)	NPV (%)	Agreement (%)	Kappa
Rifampicin	Resistant	6	1	85.7 (42–99)	100 (90–100)	100	97.8	98.1	0.912
	Sensitive	0	45						
Isoniazid	Resistant	7	1	87.5 (46.6–99.3)	100 (90–100)	100	97.7	98.1	0.922
	Sensitive	0	44						

The results showed that NRA and conventional PM do not vary significantly (*p* > 0.05) for both the drugs. The total time taken for the results of DST was calculated and was found that NRA took an average of 19.3 days and LJPM took an average of 72 days. There was a statistically significant difference in the TAT of two methods (*p* < 0.001). Out of 52 samples, 43 (82.7%) specimens yielded results in 3 weeks while 100% specimens detected results within 4 weeks’ time.

## Discussion

In this study, NRA detected growth in 53 sputum samples with the sensitivity of 91.4% while the sensitivity of LJ medium was 89.6%. Similar results have been reported by Satti et al. who obtained the high sensitivity of NRA 98.2% [9]. The high sensitivity of NRA in our study could be due to the composition of the media which contains a base supplemented with oleic acid-albumin for the rapid and luxuriant growth of Mycobacterium [15].

This study reported high sensitivity and specificity of NRA in the detection of RIF resistance (sensitivity, 85.7%; specificity, 100%) and INH resistance (sensitivity, 87.5%; specificity, 100%). The results by NRA were obtained much earlier (19.3 days) than the conventional PM (72 days) where majority (82.7%) of the positive results was available within 21 days. In contrast to our results, Satti et al. and Lamsal et al. have reported high sensitivity of 100% for RIF and more than 97% for INH [9, 16]. However, our findings were consistent with the results of majority of the studies where NRA was done directly on sputum samples [17–19]. The rapid results shown by NRA might be due to the fact that it detects the growth of MTB by detecting the color change in the medium rather than the visual detection of the colony [18]. Also, these studies regarded direct NRA as a reliable and useful method for detection of MDR-TB in resource-scarce settings.

The high sensitivity, specificity, and ease of implementation demonstrate the potential usefulness of direct NRA as an appropriate method for diagnosis of MDR-TB in resource-poor countries like Nepal. This is essential because RIF and INH are the most important and valuable drugs for management of TB. NRA in 7H11 agar is simple to adopt as it can be performed with minimal skilled personnel, equipment, consumables, and inexpensive reagents [8]. It retains the exact concentrations of drugs as the medium is solidified by agar rather than by the inspissation of the egg [9]. Furthermore, NRA has a great advantage of having shorter turnaround time compared to conventional PM which requires 6–8 weeks for primary isolation prior to performing DST, thus time savings of 56 days [5]. So, the direct NRA can be used as a rapid screening tool for detecting MDR-TB in low-income countries.

However, more studies with large sample sizes are needed to further evaluate the accuracy and applicability of direct NRA as a routine method for DST of tuberculosis in laboratories with insufficient resources.

## Conclusions

This study showed that the results of direct NRA for drug susceptibility testing of *Mycobacterium tuberculosis* were similar to the gold standard method and thus can be concluded that it is reliable and significantly faster method than the conventional method, indicating its usefulness in setting like ours.

## Abbreviations

DST: Drug susceptibility testing
INH: Isoniazid
KNO_3_: Potassium nitrate
LJ: Lowenstein Jensen
MDR-TB: Multi-drug-resistant tuberculosis
MTB: *Mycobacterium tuberculosis*
NPV: Negative predictive value
NRA: Nitrate reductase assay
PM: Proportion method
PNBLJ: *para* nitro benzoic acid containing LJ medium
PPV: Positive predictive value
RIF: Rifampicin
TAT: Turnaround time
TB: Tuberculosis
WHO: World Health Organization
XDR-TB: Extensive drug-resistant tuberculosis

## References

[1] World Health Organization (2016). Global tuberculosis report 2016.

[2] Affolabi D, Odoun M, Martin A, Palomino JC, Anagonou S, Portaels F (2007). Evaluation of direct detection of Mycobacterium tuberculosis rifampin resistance by a nitrate reductase assay applied to sputum samples in Cotonou, Benin. J Clin Microbiol.

[3] Ministry of Health and Population (2015). Department of Health Services Annual report 2014/2015.

[4] Robledo JA, Mejia GI, Morcillo N, Chacón L, Camacho M, Luna J (2006). Evaluation of a rapid culture method for tuberculosis diagnosis: a Latin American multi-center study. Int J Tuberc lung Dis.

[5] Colebunders R, Bastian I (2000). A review of the diagnosis and treatment of smear-negative pulmonary tuberculosis. Int J Tuberc lung Dis..

[6] World Health Organization (2009). Global tuberculosis control 2009: epidemiology, strategy, financing.

[7] World Health Organization (2008). Policy guidance on drug-susceptibility testing of second-line antituberculosis drugs.

[8] World Health Organization (2010). Noncommercial culture and drug-susceptibility testing methods for screening patients at risk for multidrug-resistant tuberculosis.

[9] Satti L, Ikram A, Palomino JC, Martin A, Khan FA (2013). Field evaluation of the direct detection of multidrug resistant Mycobacterium tuberculosis by nitrate reductase assay on 7H11 agar. Tuberculosis.

[10] World Health Organization (1998). Laboratory services in tuberculosis control: culture.

[11] Ministry of Health and Family Welfare (2009). Revised national TB control programme manual of standard operating procedures: culture of Mycobacterium tuberculosis and drug susceptibility testing on solid medium.

[12] Martin A, Palomino JC (2012). Procedure manual: nitrate reductase assay: drug susceptiblity testing for Mycobacterium tuberculosis.

[13] Singh S, Kumar P, Sharma S, Mumbowa F, Martin A, Durier N (2012). Rapid identification and drug susceptibility testing of Mycobacterium tuberculosis: standard operating procedure for non-commercial assays: part 2: nitrate reductase assay. J Lab Physicians.

[14] Gupta A, Anupurba S (2010). Direct drug susceptibility testing of Mycobacterium tuberculosis against primary anti-TB drugs in northern India. J Infect Dev Ctries.

[15] Fonseca L, Moore DDN. Inventory of methods for Mycobacterial culture and phenotypic drug susceptibility iesting.: From the culture and phenotypic DST sub-group of the STOP TB Partnership New Diagnostics working Group; 2011.

[16] Lamsal A, Thapa B, Timilshina M (2013). Direct nitrate reductase assay for detection of drug resistance in Mycobacterium tuberculosis: rapid , simple and inexpensive method for low resource laboratories. Int J Infect Microbiol.

[17] Ikram A, Satti L, Lalani KF, Zaman G, Gardezi HAAP (2018). Evaluation of nitrate reductase assay for early detection of multi and extensively drug resistance tuberculosis in our setup. J Coll Physicians Surg Pakistanons Pakistan.

[18] Martin A, Panaiotov S, Portaels F, Hoffner S, Palomino JC, Angeby K (2008). The nitrate reductase assay for the rapid detection of isoniazid and rifampicin resistance in Mycobacterium tuberculosis: a systematic review and meta-analysis. J Antimicrob Chemother.

[19] Kammoun S, Smaoui S, Marouane C, Slim L, Messadi-Akrout F (2015). Drug susceptibility testing of Mycobacterium tuberculosis by a nitrate reductase assay applied directly on microscopy-positive sputum samples. Int J Mycobacteriology.

